# Variability in Human-Animal Interaction Research

**DOI:** 10.3389/fvets.2020.619600

**Published:** 2021-01-15

**Authors:** Kerri E. Rodriguez, Harold Herzog, Nancy R. Gee

**Affiliations:** ^1^Human-Animal Bond in Colorado, School of Social Work, Colorado State University, Fort Collins, CO, United States; ^2^Department of Psychology, Western Carolina University, Cullowhee, NC, United States; ^3^Department of Psychiatry, Center for Human Animal Interaction, School of Medicine, Virginia Commonwealth University, Richmond, VA, United States

**Keywords:** human-animal interaction (HAI), methodology, animal-assisted intervention (AAI), variability in outcomes, replication

## Abstract

The field of Human-Animal Interaction (HAI) is plagued with mixed results. Some findings appear to indicate that interacting with a companion animal is beneficial for some aspect of human health and well-being, while other research outcomes are inconclusive or even indicate the opposite. The purpose of this paper is to take a closer look at this variability in research outcomes and to provide plausible explanations and potential remedies. Some of the reasons for mixed results are likely due to the wide variety of methodologies implemented, intermittent use of standardized measures and manualized protocols, variability in human and animal participants, and limited quantification of human-animal interactions or definitions of pet ownership. Variability in research outcomes is not unique to HAI and is, in fact, not uncommon in many more established fields such as psychology and medicine. However, the potential reasons for the variability may be linked to the unique nature of HAI in that, in its' simplest form, it involves two complex organisms, a human and an animal, interacting in dynamic ways. We argue that this complexity makes research in this field particularly challenging and requires a broad spectrum of theoretical and methodological considerations to improve rigor while ensuring the validity and reliability of conclusions drawn from study results.

## Introduction

The idea that interacting with companion animals conveys health and well-being benefits to humans goes back for centuries. Empirical research on the impact of pets on people, however, dates to the 1980s (1). Among the most influential early investigations were studies reporting that pet owners had significantly lower rates of mortality following heart attacks (2) and that interacting with dogs produced decreases in blood pressure and levels of physiological stress (3). Over the last 20 years, research on the health and therapeutic implications of the human-animal bond, including animal-assisted interventions (AAI), has grown exponentially. Hundreds of papers on these topics are now published in academic journals each year, and centers devoted to the study of human-animal relationships have been established in North America, Europe, Asia, and Australia. In addition, nearly 50 educational institutions now offer undergraduate or graduate degrees in human-animal relationships (4).

In recent years, the notion that pet owners are healthier and happier than non-pet owners has gained popularity. A 2016 survey by the Human-Animal Bond Research Institute (HABRI) found that 71% of pet owners were aware of research showing that pets improve human mental and physical health. Another HABRI survey found that 97% of family doctors who responded agreed there are health benefits of owning pets. There is a mismatch, however, between the results of empirical investigations and public perceptions of the positive effects of companion on human health and well-being (5). Some studies have found, for example, that pet owners have lower rates of mortality and obesity, higher self-esteem, are happier, and have decreased blood pressure and stress levels (6). Yet other studies have found no differences in these measures. Further, some researchers have reported that pet owners are more likely to suffer from disorders such as anxiety, insomnia, depression, obesity, ulcers, and panic attacks (6).

Research on pet ownership and loneliness exemplifies variations in results of studies on the impact of living with pets on well-being. Gilbey and Tani (7) reviewed 13 studies published between 1986 and 2014 comparing levels of loneliness in pet-owners and non-owners. Using standardized psychological instruments, five of the studies found that pet owners were less lonely, seven found no differences in the degree of loneliness in owners and non-owners, and one study reported mixed results (7). Further, only one of eight studies published between 2014 and 2020 found that pet owners were less lonely (8). Four of these studies reported no difference between the groups, and two produced mixed results. When the older and newer studies are combined, six reported beneficial associations between loneliness and pet ownership, while twelve found no association between pet-ownership and loneliness.

Outcomes from studies on the efficacy of animal-assisted intervention for improving human health and well-being have also not been uniformly positive, with similarly mixed results. For example, several studies have suggested that therapy dog visits may have beneficial physiological and psychological effects on hospitalized pediatric patients (9, 10). However, in one of the largest multi-site randomized controlled trials (RCT) on the effects of therapy dog visits on pediatric patients to date, researchers found that children in outpatient cancer treatment units who received 4 months of weekly therapy dog visits did not exhibit reduced stress, reduced anxiety, or improved quality of life compared to children randomized to treatment as usual (11). Reviews of the literature have pointed out significant threats to construct validity regarding the therapeutic value of the physical animal in AAI (12) as well as the potential for inflated false positives in findings (13) that may contribute to mixed findings in the field.

### Variations in Research Results Are Common in Science

The high degree of variation found in the results of HAI research is also common in more established fields. Experimental psychology in particular has been plagued with conflicting findings. A 2015 article in Science reported the results of replication attempts of 100 studies published in three reputable psychology journals (14). Only 39% of the results of the original studies could be replicated. Indeed, the results of widely accepted findings in behavioral research have been called into question by inconsistent findings. These include the impact of nasal oxytocin administration on interpersonal trust (15), changes in female mate preferences associated with ovulation (16), and the ego-depletion model of self-control (17).

Variability in outcomes is also common in clinical medicine. For example, a recent review of studies published in three leading medical journals found that standard treatments were based on the results of nearly 400 randomized controlled trials that failed to replicate (18). Among these were hormone therapies for menopause, breast cancer screening, knee surgery, and CPR techniques. A search of the phrase “variability in outcomes” in PubMed returned 195,828 hits, with 74,977 hits when restricted to the most recent 5-year period. The published manuscripts from these searches covered a wide range of topics such as the longitudinal course of posttraumatic stress disorder [PTSD; (19)], treatment of polycystic kidney disease (20), and recovery from arthroscopic anterior shoulder repair (21), to name but a few.

Concern for persistent variation in research results across science (the “replication crisis”) was sparked by a 2005 paper by John Ioannidis titled, “Why Most Published Research Findings Are False.” Ioannidis argued that inconsistent and false findings are particularly common in research areas that have several characteristics. These include small sample sizes, small effect sizes, “flexibility in designs, definitions, and outcomes” and, finally, fields that suddenly become “hot” (22). These problems are characteristic of many HAI research studies. Take, for example, a recent meta-analysis of research on the efficacy of animal-assisted psychotherapy for the treatment of trauma. The researchers found that seven of the nine clinical trials in the analysis were statistically underpowered; five of them had fewer than 17 subjects (23). A meta-analysis of eleven reports on the efficacy of prison-based dog programs found that most of the treatment effects in these studies were low [average *d* = 0.15; (24)]. Finally, HAI falls into Ioannides' “hot field” category. According to a Google Scholar search using the term “therapy dog,” the annual number of published papers related to canine-assisted therapies jumped from 60 in 2010 to 237 in 2019.

## Variability in Methods and Measurement in HAI Research

A specific consideration in explaining outcome variance in HAI research is that studies significantly vary in their methodological design and rigor. HAI researchers use a wide variety of designs to answer comparable questions in the field, ranging from case studies, single-subject research, and qualitative interviews to observational, cross-sectional, and longitudinal studies. While early studies in HAI were largely limited by a lack of control conditions and small sample sizes, more recent studies have substantially improved in their methodological rigor (25). Despite recent advances in methodology, systematic reviews of both animal-assisted intervention and pet ownership studies repeatedly state that is difficult to draw definitive conclusions from the data due methodological weaknesses across studies [e.g., (26, 27)].

An emerging number of studies using randomized clinical trial designs have shown promise in legitimizing the validity and strength of evidence in the field. However, even conclusions from RCTs can be limited by a high risk of bias from inadequate concealment and blinding [e.g., (28)]. Even the most rigorous studies can also vary widely in their control or comparison conditions. For example, intervention studies may feature no control at all, an active control (e.g., interaction with toys or stuffed animals), or a no-treatment control (e.g., waitlist or withdrawal periods). With a variety of control and comparison conditions used in the field, this leads to both variability in outcomes as well as difficulty making cross-study comparisons. In a systematic review of eight RCTs evaluating the effects of AAI on psychosocial outcomes, several different comparison conditions were identified including treatment without an animal present, active comparisons with human visitation or quiet reading, and waitlist controls (29). The type of control condition used may have direct impacts in study results. For example, a moderator analysis conducted as part of a meta-analysis on the effects of AAI in medical settings found that studies with a social control condition (i.e., featuring human interaction but not animal interaction) had significantly smaller effect sizes than studies with a non-social control condition (30).

Additionally, there is widespread variability in measurement methods in HAI. Specifically, the use of standardized, validated measures to quantify outcomes has been inconsistent across HAI research (25). In a systematic review of 48 studies assessing AAI in the form of reading to therapy dogs among school-aged children, only 13 studies used standardized measures with established validity and reliability to measure outcomes (31). Rather, many studies incorporate subjective ratings, researcher-created measures, or modified existing measures which makes it difficult to compare findings across studies. Still, among studies that do incorporate standardized measures, the sheer number of measures available to quantify the constructs of interest to HAI research (i.e., mental health, social functioning, quality of life) has resulted in further variability in the literature. For example, a systematic review of 14 studies on the efficacy of AAI for children with autism found that no two studies used the same standardized assessment tool (32). This lack of replication of measurement across studies prevents the ability to make informed conclusions with meta-analytic methods, which is crucial for providing an evidence base for the field (33).

Standardized measures also vary in the appropriateness of content for the theoretical outcomes of HAI. For example, a popular scale of loneliness called the UCLA Loneliness Scale was recently evaluated for its appropriateness to quantify beneficial social effects of pet ownership (34). Both qualitative and quantitative evaluation suggested that only 6/20 items were likely sensitive to change following pet ownership or pet acquisition, concluding that despite its widespread use the scale lacks efficacy for quantifying the effect of pets on loneliness. Therefore, while the use of consistent measures across studies is important for replication purposes, measures must be chosen for their sensitivity to change following animal interaction.

## Variability in Human Participants in HAI Research

When quantifying the role that animals play in our lives, it is important to consider the heterogeneity in how humans may perceive, respond to, and interact with animals. Individual differences in demographic variables such as age, gender, and race/ethnicity may contribute significantly to outcome variance. For example, a meta-analysis of outcomes from AAT found that studies of young children had the most consistently positive outcomes, while other age groups exhibited more variability in outcomes (35). Not only may males and females have different hormonal responses to interaction with animals (36), but females have been found to report more positive behaviors and attitudes toward animals (37) and toward animal-assisted interventions in general (38). While these gender differences in attitudes and responses may not be unique to HAI, equal care should be taken to consider gender-specific effects in analyses as in other fields of research. Ethnicity, cultural, and religious differences may also contribute to attitudes and perceptions of animals (39, 40), However, neither demographic variables nor other potentially confounding variables such as marital status, sources of social support, and socioeconomic status are consistently controlled for in HAI studies (41). The omission of key explanatory variables in analyses can lead to invalid conclusions if unmeasured confounding variables are partially or fully explaining significant findings. For example, a recent systematic review of the impact that pets have on child and adolescent development found that 14 of 22 studies did not consider any confounding variables in analyses, leading authors to conclude that no firm conclusions can be drawn from the literature (42). In addition to controlling for these confounders, future large-scale research studies should also consider the extent to which demographic or contextual variables may mediate outcomes (43). Mediator and/or sub-group analyses may also aid in understanding for whom and under what conditions individuals benefit from HAI (5, 44).

In addition to demographic and environmental variables, human participants in HAI research often vary widely in their physical and mental health. As a key research question in this field is understanding how animal interaction may benefit individuals of sensitive populations, HAI research often includes a range of disabilities, disorders, and chronic conditions. Frequently, participants are selected for participation in research based on a single diagnosis (e.g., posttraumatic stress disorder, cerebral palsy, etc.). However, not only is there variation across studies in how and when this diagnosis was made, there can also be considerable phenotypic variation among individuals with the same condition (e.g., autism spectrum disorder, ASD). In a systematic review of 13 studies addressing the impact of AAI on social behaviors of children with ASD, nine different terms were used to describe participants' diagnosis and/or severity including autism spectrum disorder, autism, autistic disorder, moderate autism, early childhood autism, and atypical autism (45). Thus, it is difficult to compare results across these studies when participants' symptoms and behavioral profiles are markedly different. Even in phenotypically similar disorders, there is also often participant variability in severity, progressiveness, and duration of the condition or disability. However, these factors are often not controlled for or considered in statistical analyses. For example, a systematic review of the effects of AAI on individuals with dementia found that only 13 of 32 studies controlled for the severity of dementia in their design or analysis (26). Disability severity and progressiveness can be important explanatory variables in psychosocial outcomes such as quality of life, however. In a 2006 study of the psychosocial effects of mobility service dogs for their handlers, having a progressive condition (e.g., muscular dystrophy, multiple sclerosis) was an important moderator of whether having a service dog was associated with higher positive affect (46).

Emerging research also suggests that human genetic differences may play a key role in the study of human-animal interactions. A recent 2019 study indicated that there may be a genetic and heritable component for choosing to have a pet (47). Specifically, researchers examined pet dog ownership among over 35,000 pairs of twins in Sweden and found that more than 50% of the variability in whether an individual owned a dog at the time of the study was explained by genetics. Although the specific genes associated with dog ownership could not be identified, this research suggests that a combination of environmental and genetic influences could influence an individual's affinity toward animals. Genetic variability is also an important consideration in research incorporating hormones and/or neuropeptides such as oxytocin and cortisol. Variations in the oxytocin receptor gene have been associated with human attachment behavior (48) and caregiving styles (49), and recently have been demonstrated to be associated with dog-owner attachment (50). Similarly, there are many sources of genetic and environmental influence on cortisol synthesis, metabolism, and reactivity (51, 52).

Finally, it is also important to consider differences in human experiences, thoughts, and behavior that may contribute to variable outcomes from HAI. Research suggests that interactions and relationships with companion animals can be impacted by human personality traits (53, 54). In addition, human attachment styles (e.g., avoidant or anxious attachment) can be important in understanding variation in the human-animal bond. For example, studies have shown that pet owners with avoidant attachment to their pets experience less stress-reducing benefits from their pets (55) and report negative expectations about a pet's availability and responsiveness (56). Quality and quantity of previous animal interaction, which is often unaccounted for in HAI research (5), is another important aspect of inter-participant variability. Future HAI research should be mindful of these differing experiences, including previous and current pet ownership as well as any fears or aversions toward animals, in both the design and analysis of studies.

## Variability in Animal Participants in HAI Research

Not only is there unique variation in human participants that needs to be accounted for, but also in animal participants. Animals' temperament, personality, training, and even physiology are becoming increasingly important considerations in understanding variability in HAI research. Of course, there is wide variability in the species of animals studied in this field (e.g., mammals, birds, exotics, farm animals) that contributes to heterogeneity across studies (57). However, even within a single species, there is also variability in animals' appearance, disposition, rearing/training, and history of human interaction that may influence outcomes (58). As the animal itself is a key component of HAI, detailed descriptions and considerations of animal characteristics are critical to disentangling potential mechanisms of benefits (12). In the case of AAI, a consideration of animals' varying qualities also parallels the increasing acceptance of animals as individual agents rather than tools or objects (59, 60). Researchers should also be mindful of the fact that the animal's handler during an AAI session will also vary in their experience and knowledge regarding animal welfare as an additional source of variation (61).

As one of the most commonly studied companion animals in HAI research, dogs in particular exhibit a wide range of characteristics that contribute to variability. With a variety of breeds and sizes of dogs incorporated into companion, therapy, and assistance roles, individual differences in dogs' morphology and disposition are important aspects of variation in HAI literature. For example, physical traits such as a dog's size, coat, eye color, and ear shape have been shown to impact the way that humans perceive dogs (62, 63). In addition, different breeds of dogs can significantly differ in their temperament and behavior (64, 65). For example, some breeds may be more likely to make spontaneous eye contact (66), follow human communicative gestures (67) and be more sociable or playful with humans (68) than others. Even dogs of the same breed category can differ in personality characteristics including playfulness, curiosity, and sociability (69). These individual differences may impact the way that a dog, whether in a pet, therapy, or assistance role, interacts with and potentially bond with humans in the short-term or long-term (70). In the case of pet dogs, studies have found that owners of large dogs spend more time walking their dogs (71) and engage in more training and play with their dogs (72) while small breeds are reported to have more behavioral problems (73). In fact, considering breed-specific variation in analyses is an essential step toward understanding how genetic, physical, and behavioral differences in dogs may explain or predict human therapeutic outcomes. For example, a recent study tracking over 180,000 heart attack victims and 150,000 stroke victims found that dog owners had a lower risk of mortality than non-dog owners (74). However, results varied when considering the breed and size of dogs. Owning a pure-bred retriever breed, for example, was associated with a 40% decrease in mortality rates among the heart attack victims, while owning a companion/toy breed or mixed breed dog had no association with a reduction in mortality (75).

Emerging research has also quantified how differences in dogs' physiological profiles can influence the underlying therapeutic mechanisms of action during HAI. For example, a recent study showed that a population of service dogs selectively bred for friendly and non-aggressive temperaments had higher circulating levels of oxytocin, a neuropeptide involved in human-canine social interaction and bonding, compared to pet dogs who were not selectively bred (76). Other studies have found that dogs' variation in their oxytocin receptor gene is related to certain breeds of dogs' social behavior when greeting unfamiliar people (77, 78) as well as dogs' attachment behaviors directed to their owners (50). As the oxytocin pathway has been discussed as a potential mechanism underlying positive human-dog interactions (79, 80), these individual differences across dogs may be important for understanding variability across studies.

## Variability in Human-Animal Interactions

Thus far, we have discussed variability in both human and animal participants that may contribute to observed variability across HAI research findings. However, one of the most truly variable aspects of this research lies in the nature of the human-animal interactions themselves (i.e., the physical, emotional, and/or psychological interactions that a human and an animal share). In research quantifying the benefits of pet ownership, a specific challenge lies in defining “ownership” and accounting for the variability surrounding this term (81). For example, the human-animal relationship and its resulting effects may differ between those who provide a caregiving role to the animal and those who simply cohabitate with the animal. Dogs, in particular, may also fill several different roles across households including serving as a companion, a surrogate child, or strictly for tasks such as hunting or guarding (82). It is similarly important to consider the varying length of cohabitation time and how much time a human-pet dyad spends together on a daily basis– both of which may have a significant impact on outcomes (83). Not only does the quantity of time have implications for research, but so does the quality of the interactions between an individual and a pet. For example, there is complex variation in daily dog-owner interactions that may contribute to the strength of the human-animal bond ranging from co-sleeping to frequency of cooperative activities such as play or training [e.g., (84, 85)]. Dogs have also been found to form unique attachment relationships to their owners [e.g., avoidant or anxious; (86)] that may be impacted by their owner's caregiving and/or own attachment styles [e.g., (87)]. These sources of heterogeneity have prompted researchers to use a dyadic approach to consider both the attributes of pets and owners to holistically evaluate human-animal relationships (88).

In research assessing outcomes from AAI, interactions can vary widely in terms of activities (e.g., structured or unstructured), setting (e.g., hospital bed, classroom, outdoors), human to animal ratio (e.g., group or individual interaction), and human-animal contact (e.g., duration of petting, talking, or walking). Because of this considerable variation, there is a critical need for manualized protocols and/or detailed reporting of procedures and interactions across studies (89). This will allow for a greater understanding of the benefits from AAI are due specifically to the animal's presence or to other aspects of the intervention such as novelty, attention, or human interaction (12).

During AAI, not only do the components of the interaction contribute to variability, but so does the “dosage” of the intervention in terms of total time spent interacting with an animal. For example, in a systematic review of the effects of AAI for individuals with dementia, the duration of contact with the therapy animal spanned from three, 10-min interactions in one study to bimonthly interactions over 2 years in another (26). In addition, details regarding intervention length, frequency, and content are sometimes not reported. A systematic review of AAI for trauma found that while most articles reported some aspects of the procedures surrounding the participant-animal interaction, not a single article provided enough detail to allow for replication (90). When comparing findings across studies, the omission of these critical details makes it impossible to determine the potential sources of methodological variation. Therefore, it is important for researchers to provide sufficient detail surrounding the characteristics of human-animal interaction, especially in AAI studies, to address this source of variability.

## A Discussion of the Variability Explained by the Unique Nature of HAI

There is inherent variability linked to the unique nature of HAI, in that, in its simplest form, it involves two complex organisms, a human and a companion animal, interacting in dynamic ways. Not only does HAI research need to account for human psychological, sociological, physiological, and economic variability across humans, but these same variable characteristics apply to the animal as well. For example, a person with financial resources, time, and or motivation to do so, may provide excellent veterinary care, high-quality nutrition, and opportunities for life enrichment to their companion animal that another person with fewer financial resources, less available time, or less motivation may provide to the same species of companion animal. It is important to note that this example is not intended to imply that wealthy people are better caretakers of their companion animals, but rather that each of these variables (financial resources, time, and motivation) are likely to play a role in the care and life enrichment of companion animals that will contribute to variability in pet ownership research. Further, developmental changes must also be taken into consideration. Not only will children interact differently with companion animals than adults and older adults, but each developmental stage may bring a host of unique needs or desires to human relationships with companion animals. On the other side, we cannot neglect the developmental changes taking place in the companion animals as well. Not only will animals also experience physiological, psychological, and behavioral changes as they develop, but also may gain a better understanding of their human counterparts or develop fears or aversions to humans. Therefore, not only do researchers need to keep in mind the inherent variability of the unique nature of HAI, but also how it evolves in both humans and animals over time.

As a field, HAI is charged with understanding not one, but two complex creatures, each with their own needs, motivations, and capabilities. On the human side, individuals will vary in their experiences, attitudes, abilities, and personalities that shape the way that they perceive, interact with, and ultimately bond with an animal. The animal side of the equation is further complicated by multiple species, each with different species-specific behaviors, welfare needs, physical and mental capabilities, housing and enrichment requirements, and zoonotic disease concerns. Even within the same species, there is immense additional variability in the individual (e.g., breed, temperament, personality, and behavior) that will influence its relationship and interactions with humans. Therefore, research in this field must be mindful of all of these complexities, each of which contribute to the multifaceted nature of human-animal interaction.

While the inherent variability in HAI research contributes to a unique complexity that makes for an interesting field of study, it also makes the field particularly challenging. In particular, a broad spectrum of theoretical considerations is required to account for the variability in the human, the animal, the types of interactions possible, the dynamics of the actual interaction, and any potential constraints imposed by the setting of the interaction (e.g., educational, healthcare). To achieve this, an equally broad spectrum of research methodologies must be incorporated to capture the subtle nuances of the interactions (e.g., qualitative methods) and to tightly control as many aspects of the interactions as possible (e.g., experimental methods).

## Conclusion and Future Directions

In conclusion, we have described how variability in methods and measurement, human participants, animal participants, and interactions may contribute to mixed findings in the field of human-animal interaction. We have also made suggestions on how to address this variability by using appropriate experimental designs and/or statistical analyses to account for confounding variables, by ensuring detailed reporting of both human and animal characteristics, and by providing thorough descriptions of the duration, context, and structure of human-animal interactions including replicable and/or manualized AAI procedures when possible. However, we have also discussed the inherent complexity of HAI in that even the simplest research study involves considering the dynamic interaction of two complex beings, an animal and a human.

To address the complexity of the field of HAI, researchers must face a variety of theoretical and methodological considerations to account for multiple sources of variability and individual characteristics on both the animal and human level. However, the basic tenets of science apply regardless of the complexity of the topic under study. The field of HAI demands a wide variety of methodologies and measurement, each of which provides important and useful information on which to build the field. However, whatever the approach, the experimental design must be appropriate for the research question and conclusions drawn must be mindful of limitations, including unaccounted for variability that may impact or contextualize findings. It is also incumbent upon researchers to report all results, even nonsignificant findings, as understanding the individuals, contexts, and conditions in which HAI is not beneficial is equally important for the progression of the field.

Although the field of HAI has been characterized by mixed findings, there is a wealth of promising information available on which to expand. With the growth of research in this field, new frameworks continue to emerge to study the relationships between humans and companion animals such as the dyadic approach (88), trans-species methodology (91) and the biopsychosocial model (Gee et al., under review). Inspiration from other fields, such as social psychology (92) developmental psychology (93, 94) and social neuroscience (95), will also continue to inform the theoretical underpinnings of human-animal interactions. The field will continue to benefit from an accumulation of rigorous science while building viable and testable theories. With increased funding opportunities from both public and private sources, knowledge regarding the potential therapeutic outcomes from animal interaction will continue to strengthen by incorporating randomized clinical trial designs and large-scale population studies (96). Although it is a young field, HAI has a promising foundation on which to build, and a firm commitment to scientific rigor will secure its future.

## Author Contributions

KR, HH, and NG equally contributed to the formation of the manuscript's conceptual ideas and framework. All authors contributed to writing and editing the manuscript.

## Conflict of Interest

The authors declare that the research was conducted in the absence of any commercial or financial relationships that could be construed as a potential conflict of interest.
